# Prostate cancer in Brazil and Latin America: epidemiology and screening

**DOI:** 10.1590/S1677-5538.IBJU.2015.0690

**Published:** 2016

**Authors:** Rafael Rocha Tourinho-Barbosa, Antonio Carlos Lima Pompeo, Sidney Glina

**Affiliations:** 1 Departamento Urologia, Faculdade de Medicina do ABC, Santo André, SP, Brasil

**Keywords:** Prostatic Neoplasms, Epidemiology, Early Diagnosis, Latin America

## Abstract

**Introduction:**

Prostate cancer is one of the tumors with higher incidence and mortality among men in the World. Epidemiological data are influenced by life expectancy of population, available diagnostic methods, correct collection of data and quality of health services. Screening of the disease is not standardized around the World. Up till now there is no consensus about the risks versus benefits of early detection. There are still missing data about this pathology in Latin America.

**Objective:**

to revise current epidemiologic situation and early diagnosis policies of prostate cancer in Brazil and Latin America.

**Materials and Methods:**

Medline, Cochrane Library and SciELO databases were reviewed on the subject of epidemiology and screening of prostate cancer. Screening research was performed in websites on national public health organizations and Latin America. Screening recommendations were obtained from those governmental organizations and from Latin American urological societies and compared to the most prominent regulatory agencies and societies of specialists and generalists from around the World.

**Results:**

Brazil and Latin America have a special position in relation to incidence and mortality of prostate cancer. In Brazil, it occupies the first position regarding incidence of cancer in men and the second cause of mortality. Central America has the highest rate of mortality of the continent with lower incidence/mortality ratios. Screening recommendations are very distinct, mainly among regulatory organs and urological societies.

**Conclusion:**

prostate cancer epidemiology is an important health public topic. Data collection related to incidence and mortality is still precarious, especially in less developed countries. It is necessary to follow-up long term screening studies results in order to conclude its benefits.

## INTRODUCTION

Prostate cancer is the most prevalent tumor in men, if we exclude non-melanoma skin cancers. According to GLOBOCAN data, one million and a hundred thousand men were diagnosed in 2012, corresponding to 15% of the cancers diagnosed in men (1). The incidence is quite variable around the World and influenced by life expectancy and diagnostic methods applied in each specific geographic region, as well as according to organization of epidemiological data. This is why we observe high incidence in developed regions.

**Figure 1:**
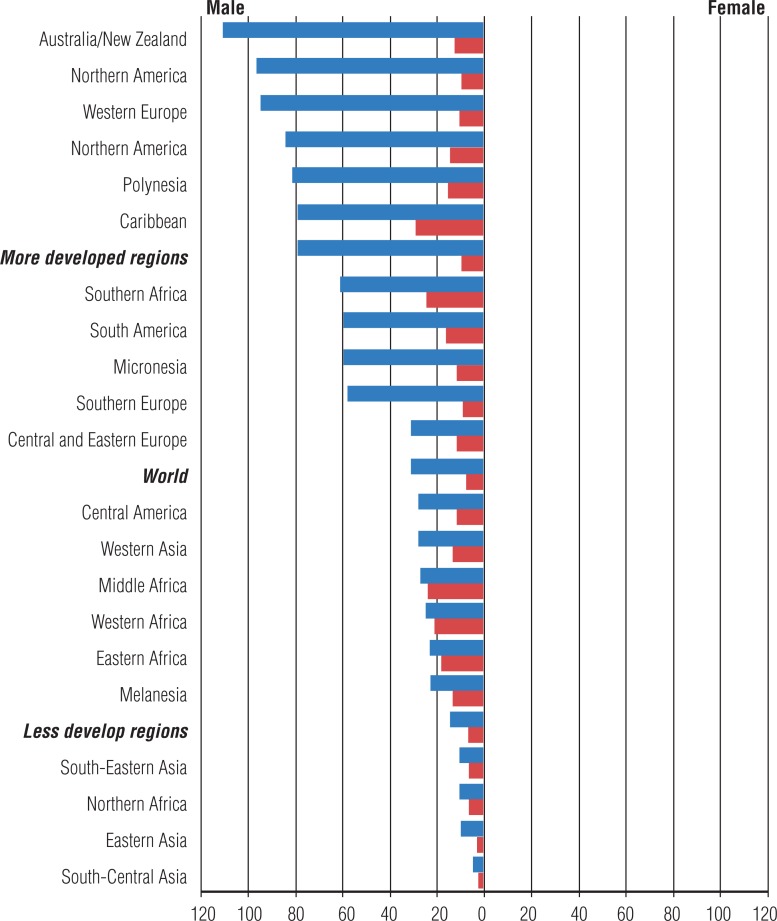
Incidence and mortality rates in World per 100.000 inhabitants source: GLOBOCAN, 2012.

In the same year of 2012, 307.000 deaths due to prostate cancer were estimated around the World, being the fifth cause of death among men (1). Mortality per region, influenced by the quality of available health services to population, is high among less developed countries. In that matter, it is observed higher incidence/mortality rates in more developed regions (Figure-1).

Early detection of prostate cancer is controversial and not standardized around the World. PSA (prostatic specific antigen) dosage is the most frequent screening method, with better cost/benefit ratio, although with limited specificity. The use of rectal exam, as part of physical exams, is influenced by culture factors among different populations.

Screening is a secondary prevention action for the detection of the disease in earlier stages. Screening programs may be population-based or opportunistic (when a patient seeks medical attention due to other reasons and the physician takes the opportunity to perform the screening).

The purpose of the screening is to detect precociously prostate cancer, to treat it in earlier phases and finally to reduce mortality. PSA false-positive results may present bad consequences to screening, including unnecessary biopsies and their complications (bleeding, infection, hospitalization) and damages due to treatment of conditions that probably would not clinically evolve (overdiagnosis and overtreatment). Overtreatment is been handled with observation strategies in very low risk diseases (active surveillance).

The results of two international randomized studies (2, 3) and their updates on the impact of screening on mortality of prostate cancer have been used to guide government health agencies, specialty societies and general medical organizations (4). Those studies were not able to demonstrate reduction of mortality after 10 years of follow-up. However, their updates show that a longer follow-up time has a tendency to treat lower number of patients in order to prevent death. Another aspect is “stage downgrade” after the implementation of early screening policies, with lower PSA, lower number of locally advanced tumors and long distant metastasis (5). In that matter, there has been great divergence among screening recommendations of government organs and medical societies.

Majority of epidemiological data and screening policies regarding prostate cancer are from studies of North America and Europe. Brazil has well defined government, non-government and medical societies policies, and in Latin America, in general, they are scarce.

## OBJECTIVE

The purpose of the present study is to review the current epidemiologic situation and health policies on early detection of prostate cancer in Brazil and Latin America, in relation to the World.

## MATERIALS AND METHODS

It was performed a search on the databases Medline, Cochrane Library and SciELO on the topics: “prostate cancer”, “epidemiology” “early diagnosis”, “screening” and their combinations, including papers in all languages. Only studies regarding Brazil and Latin America were included.

Epidemiologic research was performed in the following websites of government agencies: World Health Organization (WHO); Pan-American Health Organization (PAHO), Instituto Nacional do Cancer (INCA-Brazilian Institute of Cancer) and government health department of main countries of Latin America.

It was collected information on prostate cancer screening recommendations, and also from the following medical societies: Brazilian Society of Urology, Argentine Societies (inter-societies Consensum), Sociedad Colombiana de Urología (Colombian Urological Society), Sociedad Peruana de Urología (Peruvian Urological Society) and Sociedad Mexicana de Urología (Mexican Society of Urology).

Latin American recommendations were compared worldwide to those of government and medical societies: U. S. Preventive Service Task Force (USPSTF) - USA, National Health Service (NHS) – United Kingdom, Canadian Task Force on Preventive Health Care (CTFPHC) – Canada; American medical societies: American College of Physician (ACP), American Academy of Family Physician (AAFP); and specialties societies (American and European): American Cancer Society (ACS), American Urological Association (AUA), European Association of Urology (EAU).

## RESULTS

### Epidemiology

**Figure 2:**
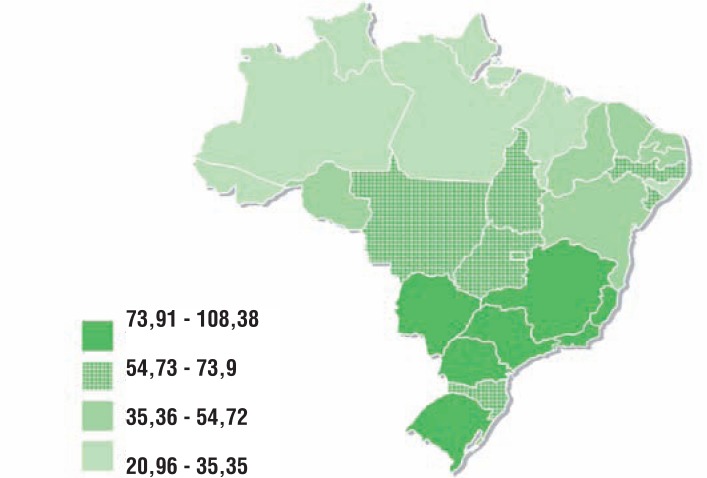
Crude rates of incidence of prostate cancer, per 100.000 men, estimated for the year 2014, according to Federation Units.

Source: INCA. Estimate 2014: Cancer incidence in Brazil

Prostate cancer is the main cancer in Brazilian men, excluding non-melanoma skin cancer. In 2012, there were 60.180 new cases, corresponding to 62 new cases/100.000 men. The most developed regions of the country registered the highest number of cases: 78 new cases/100.000 men in Southeast [40] (Figure-2). The increasing incidence of prostate cancer has been correlated to the increasing life expectancy of Brazilian population, to better assessment of medical data, to higher availability of diagnostic methods and overdiagnosis due to screening policies. There are conflicting data regarding prevalence per race: some studies show higher prevalence in African-american population and others don't demonstrate significant difference (6, 7).

**Figure 3:**
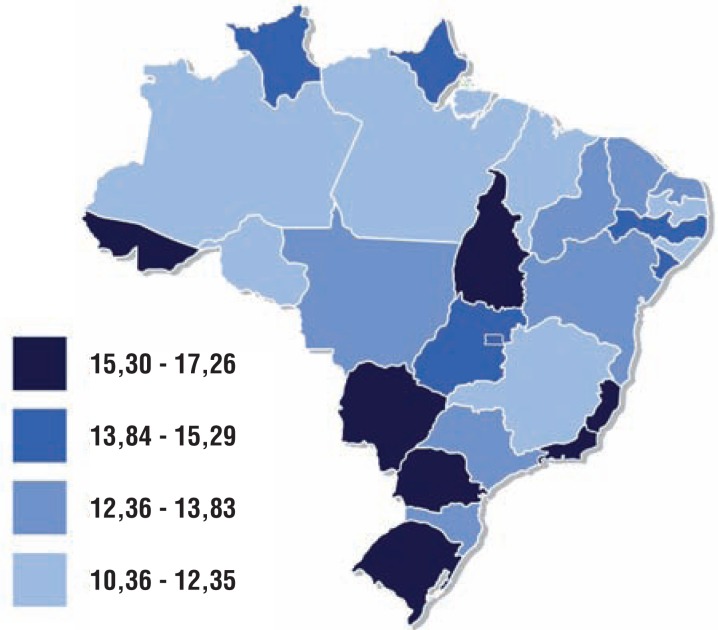
Mortality rates of prostate cancer, per 100.000 men, adjusted to World population. Brazil, 2012.

Source: INCA. Atlas of cancer mortality

In relation to mortality due to cancer in Brazilian men, prostate cancer is the second main cause of death, following lung cancer (8). In 2012, there were 13.354 deaths due to prostate tumor in Brazil, corresponding to 13% of all deaths due to cancer in men. Among these, 88% occurred in men over 65 years old (4). Figure-3 presents the distribution of prostate cancer mortality according to Brazilian regions in 2012.

Between 2004 and 2007, Hospital do Cancer in Barretos, São Paulo, Brazil, performed a huge program of prostate cancer screening in 231 Brazilian cities. Seventeen thousand patients over 45 years old were screened by plasma PSA and rectal exam. Among these, only 29% have been previously screened for prostate cancer. Biopsy was indicated in 16.1% of evaluated patients (9). Cumulative rate of prostate cancer detection was 3.7%, similar to what was published regarding North American and European populations (2, 3, 9).

The Brazilian Society of Urology published in 2012 (Nardi et al., 2012) the profile of patients assisted by public and private institutions in Brazil (10). Around 54% of prostate cancer patients were treated in public institutions, including older men (69 years x 67 years, p<0.001), more African-americans (18.3 vs. 7%, p<0.001), with more advanced disease, higher medium PSA value (10.0 vs. 6.8, p<0.001) and higher incidence of metastatic disease (10.4% vs. 4.3%, p<0.001). Among these patients, less than 50% had been submitted to radical prostatectomy, and a high proportion of patients were using hormonal ablation (chemical or surgical) combined or not to radiotherapy.

In a study performed by the Cooperative Brazilian Uro-Oncology Group (CBUG), Tobias-Machado et al. evaluated the association of level of education (schooling), screening and aggressiveness of prostate cancer in Brazil (11). Among illiterate patients, there was a lower rate of screening, and following positive screening, a lower rate of follow-up until definite diagnosis. These patients also showed higher levels of PSA, more advanced stages of the disease and higher Gleason score at biopsies.

**Figure 4:**
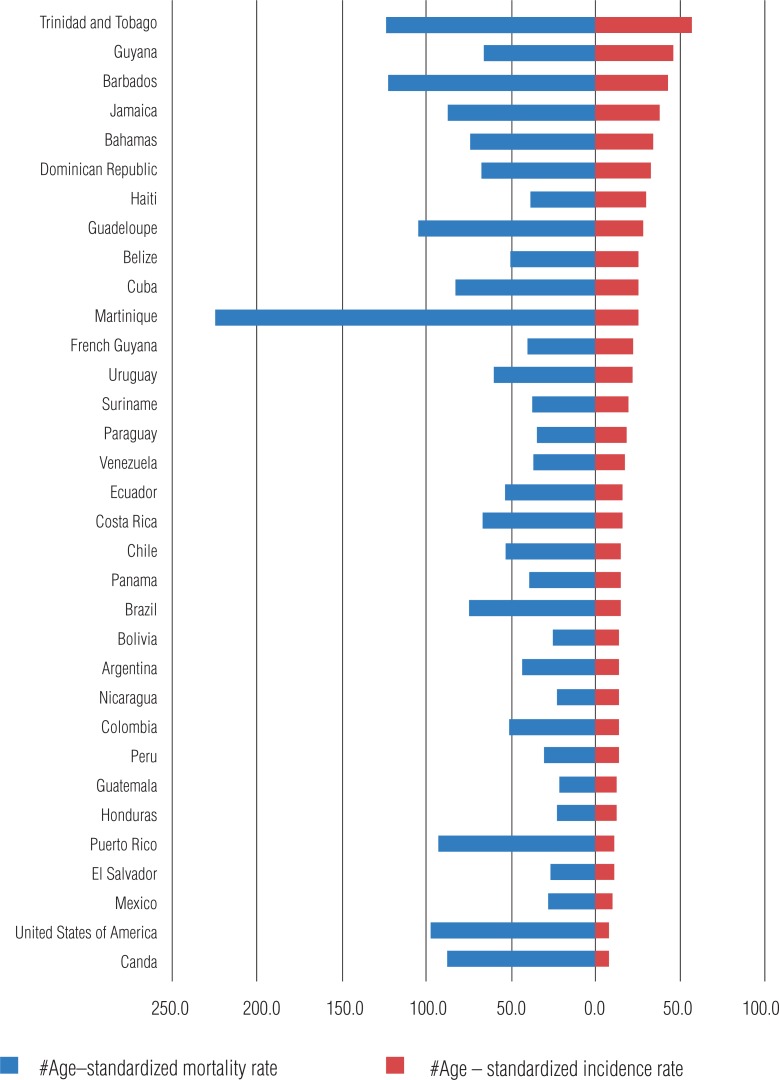
Incidence and mortality rates of prostate cancer in each American country, 2012.

Source: GLOBOCAN, 2012

**Figure 5:**
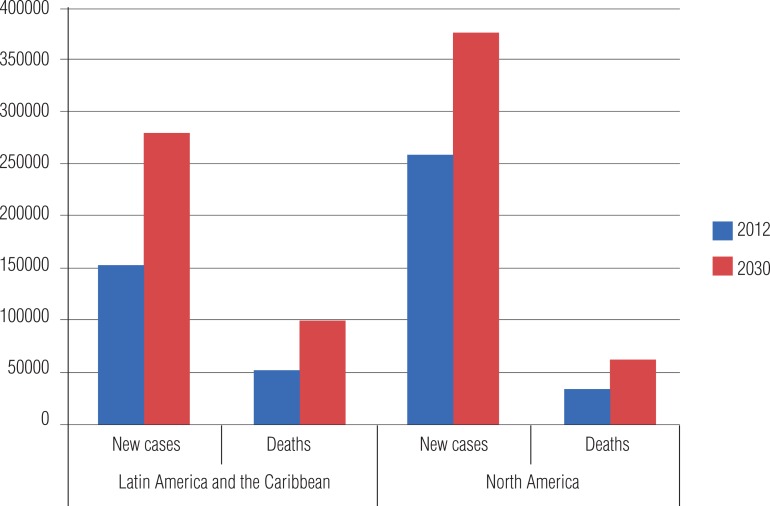
Estimate of new cases and deaths due to prostate cancer in 2012 and 2030, in the Americas.

Source: GLOBOCAN, 2012

According to data of PAHO in 2013, more than 400.000 new cases are detected per year in Latin America, with higher incidence in Central America (8) (Figure-4). It is observed an increasing incidence in Latin America, and it is expected to double by the year 2030 (Figure-5). In more developed countries, such as USA and Canada, although there is a high incidence related to early diagnosis and high quality of information systems, their mortality rates are the lowest in the continent, with high incidence/mortality rate.

**Figure 6:**
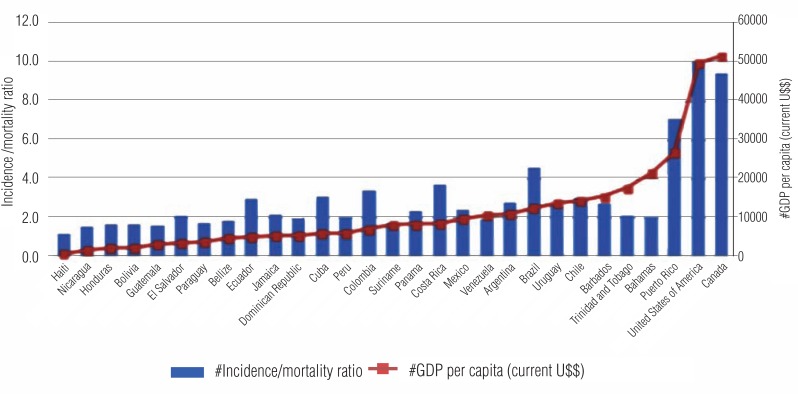
Incidence/mortality ratio of prostate cancer, compared to gnp per capita per country, 2012.

Source: GLOBOCAN, 2012

Mortality in America is still high, with 80.000 new cases per year, being the second most frequent cause of death due to cancer in men (8). These numbers are higher in Central America, followed by South and North America. There are higher rates of incidence/mortality in countries more developed and with higher Gross National Product (Figure-6).

Individual epidemiological data of different countries are scarce and few present organized and accessible data, such as Argentina and Colombia. High incidence and mortality are common in all, and prostate cancer is the second cause of death in both. Around 4.000 deaths due to prostate cancer are registered in Argentine per year and more than 2.500 in Colombia (12, 13). Mortality tendency between countries is different, as well as the health approach regarding diagnosis and treatment. In Argentine, there is a tendency to lower mortality due to better treatment in the last years (14). On the other hand, Colombia presented a recent increase in mortality (1.7% per year), related to higher diagnosis rates and better information of data (13).

### Screening

Screening recommendations for early detection of prostate cancer are divergent around the World, especially among government agencies and medical societies (Tables 1 and 2).

**Table 1:** prostate cancer screening recommendations of regulatory government agencies.

Regulatory agencies	Screening recommendations
NHS (2015) - United Kingdom	Not recommended
USPSTF (2012) - USA	Not recommended
CTFPHC (2014) - Canada	Not recommended
INCA/Health Ministry (2013) - Brazil	Organized population screening not recommended. If spontaneous demmand, inform risksxbenefits
INC/Ministerio de Salud de la Nación - Argentina	Not recommended
Secretaria de Salud - México (2010)	Patients >5y or >40a + risk factors
Ministerio de Salud e Protección Social – Colombia (2013)*	Organized population screening not recommended. Early opportunity detection if >50y or <50y + risk factors. Frequency ≥5 years.

* Ministerio de Salud and Sociedad de Urologia de Colombia recommendation

**NHS** = National Health Service; **USPSTF** = U. S. Preventive Service Task Force; **CTFPHC** = Canadian Task Force on Preventive Health Care; **INCA** = Instituto Nacional do Câncer (Brasil); **INC** = Instituto Nacional del Cancer (Argentina)

**Table 2:** Recommendations of prostate cancer screening of specialty societies.

Specialty society	Screening recommendations
American Urological Association - AUA (2013)	<40y or >70r or <10-15r de LE: do not screen
	40-54y: offer screening if with high risk^1^
	55-69y: offer screening
European Association of Urology - EAU (2015)	Men>50 years old
	Men>45 year + familial history
	African-Americans
	PSA>1ng/mL at 40 years old
	PSA>2ng/mL at 60 years old
American Cancer Society - ACS (2015)	>50 years + LE >10 yeqrs
	>45 years + high risk^1^
	>40 years + very high risk^2^
Sociedade Brasileira de Urologia - SBU (2013)	>50 years
	>45 years + high risk^1^
Consenso Nacional Inter - Sociedades (2014) – Argentina	<40y or >70y + comorbidities: do not screen
	40-55y: if with high risk
	55-70y or >70y without comorbidities: shared decision
Sociedad Colombiana de Urologia (2013)*	Organized population screening not recommended
	Early opportunity detection if >50u or <50y + risk factors
	Frequency ≥5 years.
Sociedad Peruana de Urología	>50 years
	>40 years + high risk^1^
Sociedad Mexicana de Urologia	>45 years

* Ministerio de Salud and Sociedad de Urologia de Colombia recommendations

LE = Life expectancy

1 High risk: 01 first-degree relative with prostate cancer or African-american

2 Very high risk: >01 frst-degree relative with prostate cancer

Internationally, health organizations of North America, Canada and United Kingdom are against population screening. For example, in United Kingdom, there is no screening program for prostate cancer based on PSA, according to recommendations of NHS of 2015 (15). Also, the regulatory agencies of the two biggest countries of North America, USPSTF (2012) and CTFPHC (2014) are contrary to organized population screening (16, 17).

INCA, an organ from the Health Ministry of Brazil, in 2013, also is contrary to population screening, since their risks overpass the benefits. However, in cases of spontaneous demand, the agency recommends to orientate the patient about the risks versus benefits and shared decision (18).

Accordingly, Ministerio de Salud and Instituto Nacional del Cancer of Argentine do not recommend population screening (12). In Mexico, the Secretaria de Salud (Health Secretary) is one of the few government agencies to recommend PSA and rectal exam to patients with more than 50 years old or above 40 years with risk factors (19).

Among general medical societies, AAFP (2013) follows the recommendations of government agencies, against screening, independent of age (20). Meanwhile, ACP (2013) orients to not offer early detection for patients under 50 years old, above 70 years or with life expectancy lower than 15 years, but recommends shared decision for patients between 50 and 59 years old (21).

Specialties societies present different visions of government agencies: they widely recommend screening with tiny differences regarding the candidate patients and interval. Among international societies, AUA (2013) recommends screening patients between 55 and 69 years old, and above 40 years for patients with high risk (22). ACS (2015), an oncological society, suggests discuss screening for patients with more than 50 years old, and life expectancy over 10 years or above 45 years in the presence of high risk factors (African-americans or first-degree relatives with prostate cancer) or above 40 years if with high risk (more than a first-degree relative with prostate cancer) (23). EAU (2015) also recommends screening for patients over 50 years old or with more than 45 years with risk factors; they also recommend to screen men over 40 years old with PSA higher than 1ng/mL or older than 60 years with PSA higher than 2ng/mL (24).

In Brazil, SBU strictly recommends population screening and it pioneered men education regarding the issue. In 1996, the society started educational campaigns with artists, encouraging men to screening. In 2012, the campaign “Campanha Novembro Azul” was proposed in order to stimulate men over 40 years old to seek preventive exams. In the last update of 2013, SBU claims that the target population are men over 50 years old or above 45 years if with high risk (African-americans, or with familial history of first-degree relatives) (25).

Latin American societies, as in Brazil, recommend screening with PSA dosage and rectal exam. Peruvian society recommends screening for men over 50 years old or above 40 if with high risk (26). In Mexico, the urological society recommends screening for men over 45 years old (27). National Consensum of Argentinian Inter-societies follow AUA recommendations (28). In Colombia, the Ministerio de Salud e Protección Social and Sociedad Colombiana de Urologia (Guia de Práctica Clinica, 2013), do not recommend organized population screening, but early detection by opportunity for men over 50 years old or below 50 years if with risk factors. Contrary to others, the screening interval should not be inferior to 5 years (13).

## CONCLUSIONS

Prostate cancer epidemiology by itself reinforces the impact of that disease in public health. The World is facing the disease with attitudes to lower the associated morbidity and mortality. Brazil is ameliorating the combat to that disease, adopting policies of early detection, improvement of diagnosis and treatment, but most Latin American countries do not follow this advances, also with lack of correct information. The result is the observation of high rates of mortality, mainly in Central America.

Screening policies are divergent among countries and even internally, among government agencies and medical societies. Long follow-up of large international studies will establish the importance of screening and will help define future recommendations. However, the frequent demands for implementation of screening programs must be followed by correct actions of diagnostic confirmation and treatment.
